# Relationship between CSF dynamics and outcome after shunting in NPH- 20 years of single centre experience

**DOI:** 10.1186/2045-8118-12-S1-O64

**Published:** 2015-09-18

**Authors:** Eva Nabbanja, Marek Czosnyka, Nicole Keong, Matthew Garnett, John D Pickard, Zofia Helena Czosnyka

**Affiliations:** 1 Neurosurgery, University of Cambridge, UK

## Objective

The strict relationship between shunt-responsiveness and increased resistance to CSF outflow (Rout) as reported in 1981 by Borgensen and Gjerris [1] was later presented as significant but not strong in the‘Dutch NPH’ trial [2] and recently reported as unconvincing in the ‘European NPH study’ [3]. We reviewed our ongoing database to study the relationship between parameters describing CSF circulation and pressure-volume compensation with clinical improvement after shunting.

## Method

310 adult patients (age 40-86) were included in retrospective analysis. All patients had probable NPH following clinical assessment including imaging. Patients underwent lumbar or intraventricular infusion studies and were available for follow-up via the multidisciplinary CSF clinic. Outcomes were assessed using the in-house Cambridge Outcome Scale; a pragmatic categorization of patient cohorts into three groupings – sustained improvement, short-term improvement and no improvement. Compensatory parameters were calculated on the basis of infusion test: baseline ICP and pulse amplitude, Rout, elasticity, estimator of CSF production, slope of amplitude-pressure regression line. Consultants deciding about shunting were not blind to results of infusion study.

## Results

79% of patients showed improvement (60% sustainable, 19% temporary). Improvement rate increased from 1992 (60%) to 2013 (86%); p=0.0003. Of all calculated CSF compensatory parameters, only Rout was associated with outcome (p=0.014). Pulse amplitude of ICP (peak-to-peak), slope of amplitude-pressure regression line, elasticity, did not correlate with outcome significantly. Patients with Rout>13 mm Hg/(ml/min) had an improvement rate of 79%, compared to 63% (p=0.011) with Rout<13. Notably, none of patients with low Rout (lower than 6 mm Hg/(ml/min); N=5) improved after shunting. Neither age nor sex correlated with outcome.

## Conclusion

Rout was the only CSF compensatory parameter correlating with outcome following shunting. The relationship was weak but significant. Infusion studies appeared to be helpful in the assessment of compensatory parameters both for diagnostic and to yield baseline values as a benchmark for further investigations in cases of suspected shunt malfunctions and complications.
